# The Combination Vaccine Adjuvant System Alum/c-di-AMP Results in Quantitative and Qualitative Enhanced Immune Responses Post Immunization

**DOI:** 10.3389/fcimb.2019.00031

**Published:** 2019-02-19

**Authors:** Thomas Ebensen, Simon Delandre, Blair Prochnow, Carlos A. Guzmán, Kai Schulze

**Affiliations:** Department of Vaccinology and Applied Microbiology, Helmholtz Centre for Infection Research, Braunschweig, Germany

**Keywords:** adjuvant system, combination, c-di-AMP, alum, humoral, cellular

## Abstract

The development of new effective vaccines strongly depends on adjuvants and formulations able to stimulate not only strong humoral responses against a certain pathogen but also effector as well as memory CD4+ and CD8+ T cells (Dubensky et al., 2013). However, the majority of vaccines licensed for human use or currently under clinical investigation fail to stimulate efficient cellular responses. For example, vaccines against hepatitis B virus (HBV), human papillomavirus (HPV), diphtheria, tetanus and influenza are usually administered by intramuscular (i.m.) injection and contain aluminum salts (alum) as adjuvant. Alum has been shown to stimulate Th2 immune cells resulting in increased production of antigen-specific antibodies but to be incapable of stimulating robust Th1 or cytotoxic responses. To overcome such limitations recent research has focused on the development of adjuvant combinations (e.g., MF59, AS03 or AS04) to not only further strengthen antigen-specific immune responses but to also allow their modulation. We have shown previously that bis-(3′,5′)-cyclic dimeric adenosine monophosphate (c-di-AMP) constitutes a promising adjuvant candidate stimulating both effective Th1/Th2 and cytotoxic immune responses when included in mucosal or parenteral vaccine formulations. In the present work we demonstrate that c-di-AMP can be also combined with other adjuvants like alum resulting in increases in not only humoral responses but more striking also in cellular immune responses. This leads to improved vaccine efficacy against intracellular pathogens.

## Introduction

Today, infectious diseases represent the second leading cause of death worldwide (Global Health Observatory Data Repository, 2016). In order to prevent human illness and death, vaccination is currently the most effective tool. According to the World Health Organization (WHO), vaccination prevents 2 to 3 million deaths each year. Moreover, this number could even reach 6 million, if all children would receive the recommended vaccine schedule (Patil and Shreffler, 2018). Currently, vaccination allows us to control up to 10 major diseases and has resulted in the eradication of the smallpox virus in 1980. Although vaccination has demonstrated its strength to protect against infectious diseases, the emergence of new pathogens, as well as the increase of antibiotic resistance, reveals the necessity for the development of new vaccines. Presently, the vaccines in use are based on either live-attenuated pathogens, inactivated whole pathogens (virus or bacteria) or only pure microbial components. The latter, so-called subunit vaccines, constitute promising candidates for the development of vaccines showing increased safety profiles. Since subunit vaccines contain no living organism, these vaccines are especially useful for vaccination of immunocompromised individuals. However, the low complexity profile of subunit vaccines makes them less immunogenic. Booster immunizations and/or the inclusion of adjuvants are/is therefore required. In this context, adjuvants are not only used to enhance the stimulated antigen-specific immune responses but also to tailor the immune responses according to the specific clinical needs. In this regard, it is unlikely that a single adjuvant will be able to fulfill all the required properties to be implemented in all foreseeable vaccines. Presumably, different adjuvants are needed that stimulate the immune responses required following different vaccination strategies considering the pathogen, the type of antigen, the immune status and age of the vaccine and the application route. Recent approaches also address the possibility to combine different adjuvants in order to improve vaccine efficacy (Garcon and Di Pasquale, 2017). Especially the stimulation of a cell-mediated immune [T helper 1 (Th1) response and cytotoxic T lymphocytes (CTLs)] is of interest since actual vaccines stimulate predominantly humoral immune responses (Riese et al., 2013; Lee and Nguyen, 2015; Tandrup Schmidt et al., 2016). However, despite significant progress in adjuvant development during the last decades only a limited number of adjuvants are available for human use (Di Pasquale et al., 2015). Therefore, the aim of the present work was to evaluate the potential of the STING agonist c-di-AMP to increase vaccine efficacy when combined with the well-known adjuvant alum. In order to achieve this goal immunization studies were performed using the model antigen beta-galactosidase allowing an in depth dissection of the immune effector mechanisms stimulated by this system. Alum is the most used adjuvant worldwide and represents one of five adjuvants approved in the United States (FDA, 2011). However, alum stimulates only antigen-specific Th2 immune cells resulting in the secretion of IL-4, IL-5, and IL-10 and the subsequent improved antigen-specific antibody production (Brewer et al., 1999). In contrast, the cyclic di-nucleotide c-di-AMP, a second messenger in prokaryotes, exhibits strong immune modulatory properties - stimulating antibody and mixed Th1/Th2 as well as cytotoxic responses when administered by either parenteral or mucosal routes (Ebensen et al., 2007a,b; Ebensen et al., 2017; Schulze et al., 2017a). This renders it very attractive for use in human vaccines, since most adjuvants supporting a Th1-dominated response lack the ability to induce humoral immunity (Libanova et al., 2010; Matos et al., 2017). Moreover, c-di-AMP is also able to promote the stimulation of CTL responses by induction of cross-priming (Lirussi et al., 2017). Therefore, an adjuvant system of alum/c-di-AMP could overcome the limitation of alum and not only further enhance the stimulated antigen-specific humoral response but at the same time promote the stimulation of Th1 and CTL responses.

## Materials and Methods

### Mice

Female BALB/c (H-2d) mice 6–8 weeks of age were purchased from Harlan Germany and kept at the animal facility of the Helmholtz Centre for Infection Research (Germany) under specific pathogen-free conditions as previously described (Schulze et al., 2017a). The animal experiments in this study have been reviewed for ethical compliance by the institutional ethical board and approved by the local government of Lower Saxony (Germany, No. 33.42502-13/1281). All experiments in this study were performed following standard biosecurity and institutional safety guidelines.

### Immunization Protocol

Animals (*n* = 10) were immunized 3 times at day 0, 14, and 28 by intramuscular route. Each animal received a dose of 50 μl containing 15 μg of β-Gal protein (Sigma-Aldrich, Germany) as antigen. ß-Gal was either adsorbed to alum [1:1 v/v, aluminum hydroxyphosphate (Adju-Phos^®^), Brenntag Biosector, Denmark] at pH 7.4 and 25°C or co-administered with c-di-AMP (Biolog, Germany) at a concentration of 5 μg per dose. Fourteen days after the third immunization, spleens of vaccinated mice were collected, immune cells were extracted, pooled and restimulated with β-Gal. The cytokine concentration was measured by cytometric bead array (CBA). Results from one representative out of two independent experiments are shown.

### Elisa

β-Gal-specific antibody titers in sera were investigated using ELISA assay as previously described (Schulze et al., 2017a). In brief, high binding protein plates were coated with β-Gal protein (2 μg/ml in 0.05 M carbonate buffer). After blocking unspecific binding sites using 3% bovine serum albumin (BSA) in PBS serial 2-fold dilutions of sera in 3% BSA/PBS were added (100 μl/well). After 1 h incubation at 37°C, plates were washed using 1% BSA/PBS/0.05% Tween 20 and the secondary antibodies were added: biotinylated goat anti-mouse IgG, IgG1, and IgG2a (Sigma, USA), respectively. After 1 h incubation at 37°C, plates were washed and samples were incubated for 1 h at RT in the presence of peroxidase-conjugated streptavidin (BD Pharmingen, USA). Finally, reactions were developed using ABTS [2, 20-azino-bis(3- ethylbenzthiazoline-6-sulfonic acid)] in 0.1 M citrate-phosphate buffer (pH 4.35) containing 0.01% H_2_O_2_. Endpoint titers are expressed as absolute values of the last dilution giving an optical density (OD405 nm) being two times higher than the values of the negative control after 5 min incubation as previously described (Ebensen et al., 2007b).

### ELISpot Assay

The quantity of β-Gal-specific cytokine-producing cells was investigated using an ELISpot assay as previously described (Lirussi et al., 2017; Schulze et al., 2017b). Flat bottomed 96-well plates with a 0.45 μm hydrophobic High Protein Binding Immobilon-P-Membrane (BD Pharmingen) were coated with anti-IFN-γ, anti-IL2, anti-IL4 or anti-IL17 antibodies diluted in PBS and incubated overnight at 4°C. Unspecific binding sites were blocked for 2 h at RT using 200 μl/well of complete medium. Then, 4 × 10^5^ and 2 × 10^5^ spleen cells/well were added and incubated in the absence (blank, only media added) or presence of the β-Gal protein (5 μg/ml) and the MHC-I immunodominant peptide TPHPARIGL of β-Gal (5 μg/ml), respectively. For positive controls, splenocytes were stimulated with 5 μg/ml of the mitogen concanavalin A. Samples were incubated for 16 (IFN-γ) or 48 h (IL-4) at 37°C. Afterwards, plates were washed and further incubated for 2 h at RT in the presence of appropriate diluted biotinylated detection antibodies. Then, after another washing step samples were incubated for 1 h at RT in the presence of peroxidase-conjugated streptavidin. After a final wash, cytokine-secreting cells were detected by adding AEC substrate (diluted in 0.1 M acetate buffer pH 5.0) mixed with 0.05% H_2_O_2_ (30%). After stopping the reaction with distilled water, plates were analyzed using the ImmunoSpot Image Analyzer software v3.2 (CTL-Europe GmbH). Results are expressed as Spot Forming Units (SFU) obtained from stimulated cells subtracted of background from non-stimulated cells (Ebensen et al., 2007b).

### Proliferation Assay

The ability of immune cells derived from spleen to proliferate upon restimulation with β-Gal as well as their cytokine profile were measured 96 h post restimulation. To this end, cell suspensions were seeded at 5 × 10^5^ cells/well in flat-bottomed 96-well microtiter plates (Nunc) and incubated for 4 days in the presence of 1, 10, 20, and 40 μg/ml of the β-Gal protein. During the final 18 h of culture, 1 μCi of [^3^H]-thymidine (Amersham International, Freiburg, Germany) was added to each well. Cells were harvested on paper filters (Filtermat A; Wallac, Freiburg, Germany) by using a cell harvester (Inotech, Wohlen, Switzerland) and the number of proliferating cells was indirectly determined by counting [^3^H]-thymidine events incorporated into the DNA of proliferating cells with a γ-scintillation counter (Wallac 1450, Micro-Trilux) (Lirussi et al., 2017; Schulze et al., 2017b). Results are expressed as ratio of values from stimulated and nonstimulated samples [stimulation index (SI)].

### Multiplex Flowcytomix (Cytometric Bead Array)

Supernatants of antigen-restimulated spelenocytes have been used to characterize the stimulated cytokine profiles using the Th1/Th2/Th9/Th17 FlowCytomix immunoassay from Biolegend according to the manufacturer's instructions (Ebensen et al., 2017).

### Statistical Analysis

Statistical significance of the observed differences was analyzed using the one-way ANOVA test (Tukey's multiple comparisons test) of the Graph Pad Prism 7 software for Windows (Version 7.04) as previously described (Mittal et al., 2015). Differences were considered significant at *p* < 0.05 (^*^), *p* < 0.01 (^**^), *p* < 0.001 (^***^), and *p* < 0.0001 (^****^).

## Results

### Adjuvant System C-di-AMP/Alum did not Induce any Signs of Unwanted Side Effects

**Figure 1:**
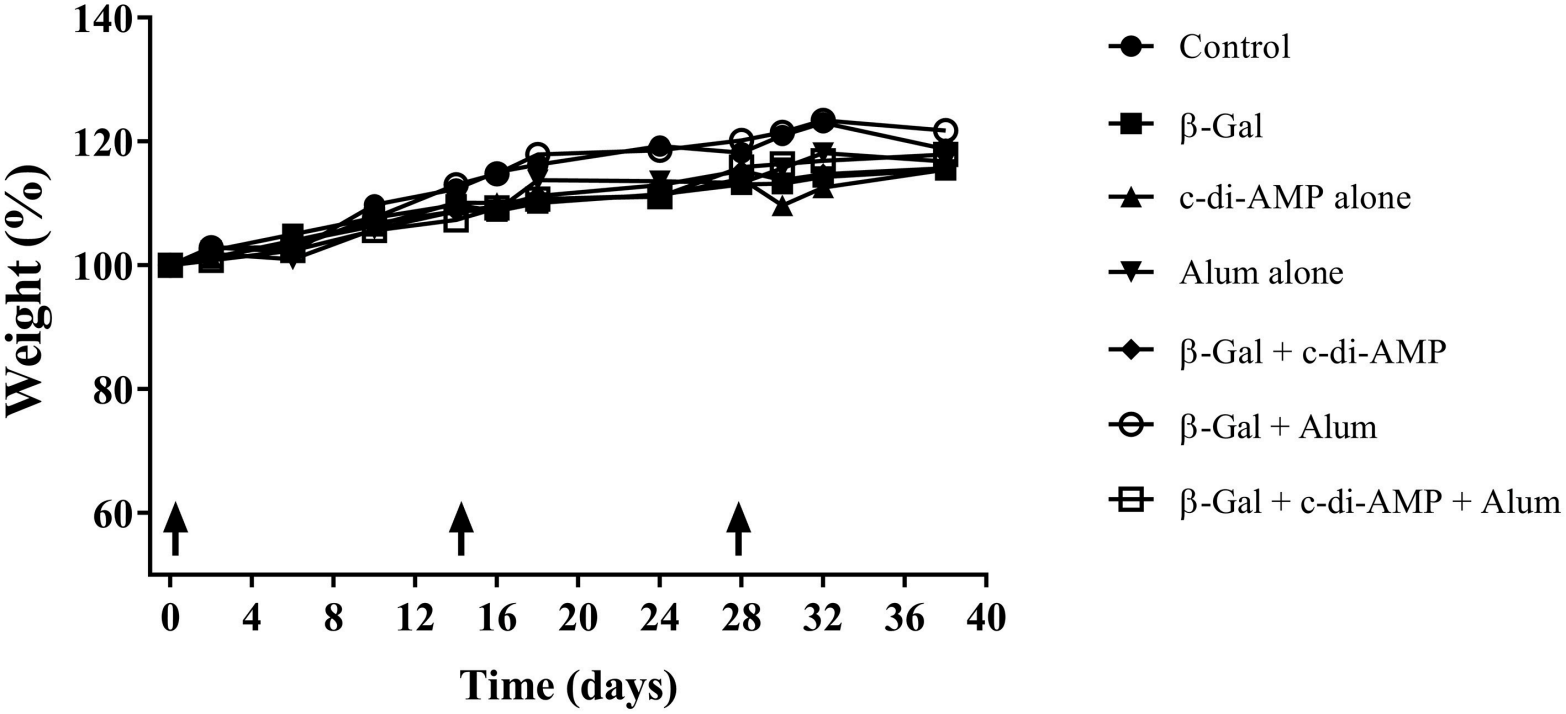
Development of the weight of mice vaccinated with different β-Gal-containing formulations. Animal body weight was monitored throughout the whole experimental setting.

The aim of this study was to investigate if c-di-AMP in combination with alum can further optimize the immune response against an antigen (β-Gal) when given by parenteral route. In a first attempt, the general behavior and the body weight development was investigated during the course of vaccination. In all vaccinated groups, no changes in spontaneous and provoked behavior, in grooming, feces character and body weight (Figure 1) were observed.

### Adjuvant System C-di-AMP/Alum Promotes Antigen-Specific Antibody Responses

**Figure 2:**
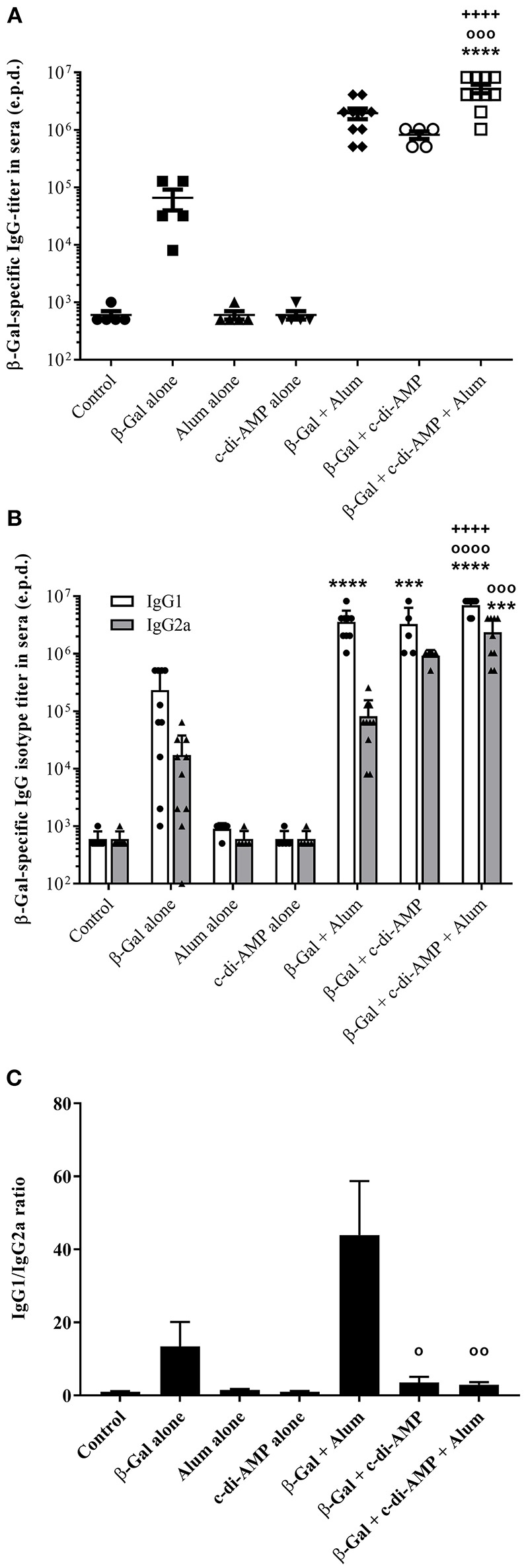
Systemic humoral immune responses induced in mice after three immunizations with β-Gal co-administered with the adjuvant system c-di-AMP/alum via intramuscular route. **(A)** β-Gal–specific IgG titers in sera 13 days after the last immunization. **(B)** β-Gal–specific IgG1 and IgG2a in the sera of immunized mice (*n* = 10). Results are displayed as average of the last sera dilution (end point dilution, e.p.d) showing the double value (OD 405 nm) of the control background. Each bullet symbol indicates a single animal, whereas horizontal lines represent the mean of animals. **(C)** β-Gal–specific IgG1/IgG2a ratio in the sera of immunized mice. Statistically significant differences were validated using the one-way ANOVA with Tukey's *post hoc* test with *p* < 0.001 (***) and *p* < 0.0001 (****) when compared with β-Gal alone (*), or in combination with alum (o) or c-di-AMP (+), respectively.

The β-Gal-specific antibody titers post third immunization with or without the combination of alum and c-di-AMP are displayed as end-point-dilution titer in Figure 2. The combination of c-di-AMP with alum led to the stimulation of a stronger humoral immune response. The antigen specific antibody titers stimulated by c-di-AMP combined with alum are higher by a factor of 2.5 and 6.4, respectively, than with alum or c-di-AMP alone (Figure 2A). Moreover, only the adjuvant combination stimulated β-Gal-specific antibody titers that were statistically significantly higher compared to the titer stimulated by β-Gal alone or in combination with any single adjuvant. The humoral immune response can also give some indications regarding the polarization of the T-lymphocytes by looking at the ratio between IgG1/IgG2a antibody isotypes (Figures 2B,C). Taken individually, alum and c-di-AMP evoke the same IgG1 titer (3.6 × 10^6^ and 3.3 × 10^6^, respectively), which was significantly higher compared to those observed in mice receiving β-Gal alone. However, the combination of alum and c-di-AMP resulted in significantly increased IgG1 titers compared to those obtained using single adjuvants (Figure 2B). Although not statistically significant, c-di-AMP alone induces an almost 10-fold higher titer of β-Gal-specific IgG2a than alum alone. Again, the combination of the two adjuvants stimulated IgG2a titers significantly increased compared to those stimulated by β-Gal alone or adsorbed to alum. Thus, the addition of c-di-AMP with alum in the formulation somehow restores the ratio between IgG1/IgG2a compared to alum alone (ratio of 2.96 for the combination and 43.21 for alum alone; Figure 2C). Moreover, the ratio IgG1/IgG2a between c-di-AMP and the adjuvant combination is unchanged (3.56 and 2.96, respectively). Thus, the alum and c-di-AMP combination leads to not only a higher IgG1 titer but also a better IgG1/IgG2a balance, which could be translated into a better balance between the Th1/Th2 immune response (Figure 2C).

### Adjuvant System C-di-AMP/Alum Promotes Antigen-Specific Cellular Responses

**Figure 3:**
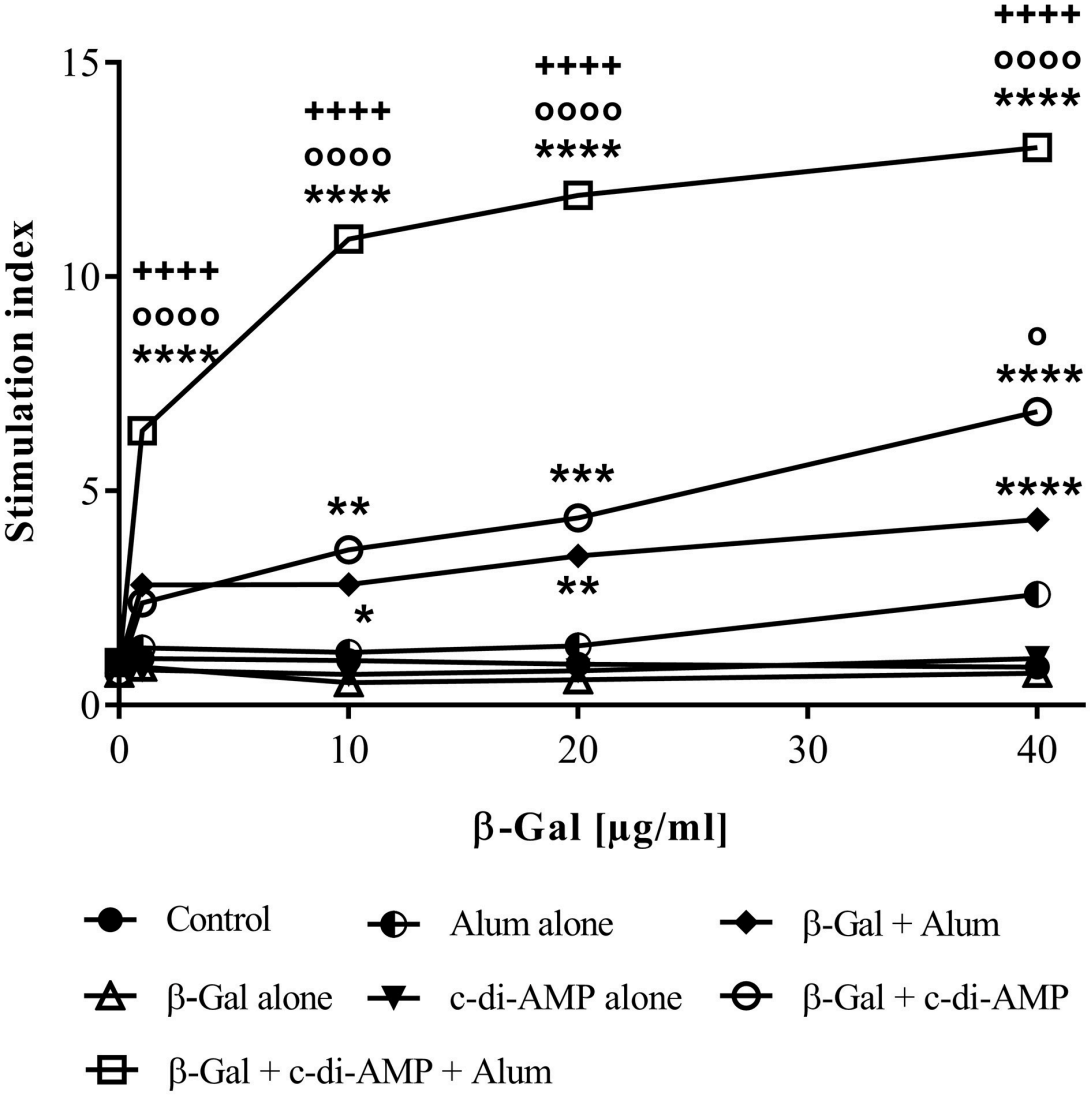
Cellular responses induced in mice after three immunizations with β-Gal co-administered with the adjuvant system c-di-AMP/alum via intramuscular route. Splenocytes from vaccinated animals (*n* = 10) were restimulated with an increased concentration of β-Gal protein (starting from 1 μg/ml to 40 μg/ml). Proliferation of the cells after 96 h was indirectly determined by counting [^3^H]-thymidine events incorporated into the DNA of proliferating cells. Results are average of quadruplicates and are expressed as stimulation index (SI). Statistically significant differences with respect to β-Gal alone (*), β-Gal with alum (o) or β-Gal with c-di-AMP (+) were validated using one-way ANOVA with Tukey's *post hoc* test, with *p* < 0.05 (*), *p* < 0.01 (**), *p* < 0.001 (***) and *p* < 0.0001 (****).

The strongest proliferative capacity upon antigen restimulation was recorded in mice vaccinated with β-Gal and both adjuvants, as shown by the stimulation index of 13 (Figure 3). The proliferative capacity of the splenocytes is not only statistically significantly higher compared to those observed in mice vaccinated with β-Gal alone or in combination with a single adjuvant but also occurs already when restimulated with a very low concentration of antigen (1 μg/ml of β-Gal), indicating a strong activation of antigen-specific immune cells. In contrast, nearly the same stimulation index is obtained only when splenocytes derived from mice immunized with β-Gal plus c-di-AMP were restimulated with 40 μg/ml of β-Gal protein (Figure 3).

**Figure 4:**
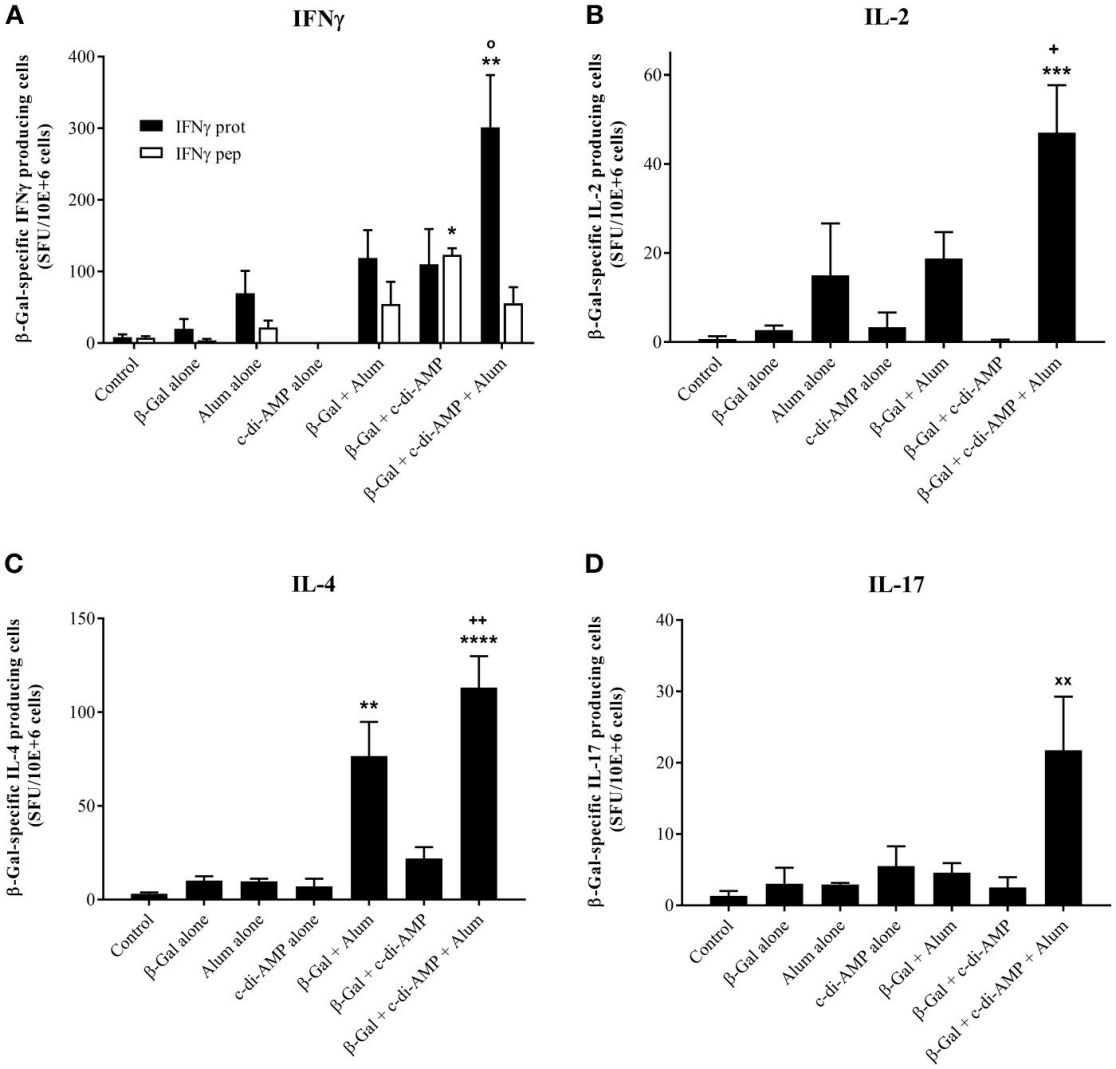
Antigen-specific cytokine-producing cells stimulated by β-Gal co-formulated with different adjuvants. The quantity of β-Gal-specific cytokine-producing splenocytes was determined by ELISpot assay. IFN-γ **(A)** IL-2 **(B)**, IL-4 **(C)**, and IL-17 **(D)** were investigated as indicator cytokines for Th1, Th2, and Th17 biased immune responses. Results obtained restimulating with the TPHPARIGL peptide reflect the number of IFN-γ producing CD8+ T cells while restimulation with β-Gal protein activates CD4+ and by cross-presentation also CD8+ T cells. Results are presented as mean spot-forming units per 10^6^ cells above the background values of unstimulated cells. The SD were calculated from triplicates of two cell concentrations each of control, antigen alone, co-administered with c-di-AMP, alum or adjuvant system c-di-AMP/alum. Statistically significant differences with respect to Control (x), β-Gal alone (*), β-Gal with alum (o) or β-Gal with c-di-AMP (+) were validated by one-way ANOVA with Tukey's *post hoc* test, with *p* < 0.05 (*), *p* < 0.01 (**), *p* < 0.001 (***), and *p* < 0.0001 (****).

Upon *in vitro* antigen restimulation, lymphocytes of vaccinated animals do not only proliferate, they also secrete cytokines which for some are the hallmark of their polarization. As represented in Figure 4, immunization with the combination of alum and c-di-AMP allows the stimulation of a significantly higher number of β-Gal-specific IFN-γ-producing cells than with alum alone. The combination seems also to be more potent than c-di-AMP alone, however, the observed difference wasn't significant. In contrast, when investigating the number of antigen-specific IFN-γ-producing CD8+ T lymphocytes, their number seems to decrease following vaccination with β-Gal using both adjuvants (the average number is comparable to alum alone) as indicated by the levels of β-Gal-specific IFN-γ-producing cells observed when splenocytes were restimulated with the CD8 peptide TPHPARIGL (Figure 4A). Thus, the elevated levels of IFN-γ-producing cells observed when splenocytes of mice receiving the adjuvant system c-di-AMP/alum were restimulated with β-Gal protein seem to be CD4+ T cells. The number of IL-2, IL-4 and IL-17 β-Gal-specific-producing cells is also statistically significantly increased in mice immunized with the adjuvant system c-di-AMP/alum with respect to those detected in mice receiving β-Gal alone. This supports the assertion of a more balanced T helper response with respect to the formulation encompassing only single adjuvants (Figures 4B–D).

**Figure 5:**
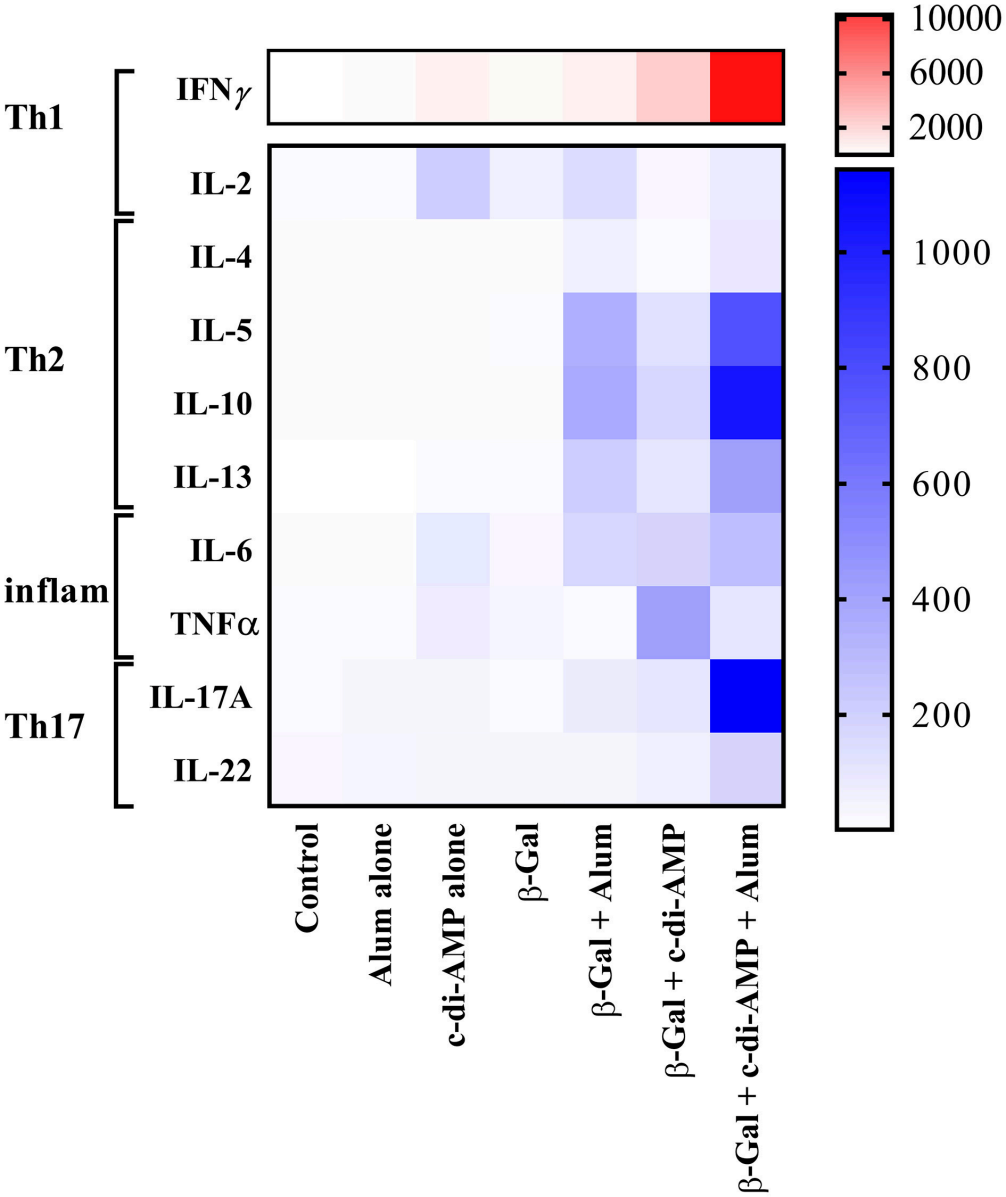
Cytokine profiles stimulated by β-Gal co-formulated with different adjuvants. The presence of mouse IL-2, IL-4, IL-5, IL-6, IL-10, IL-13, IL-17A, IL-22, IFN-γ, and TNF-α were determined in an immunoassay using a cytometric bead array according to the manufacturer's instructions (Mouse Th1/Th2/Th9/Th17 13plex Biolegend). Results are presented as a heat map of Th1, Th2, pro-inflammatory, and Th17 cytokines in antigen-restimulated splenocytes derived from mice (*n* = 10) vaccinated with β-Gal co-administered with alum, c-di-AMP or c-di-AMP/alum.

The results obtained analyzing the number of cytokine-producing cells were in line with the levels of the corresponding cytokines detected in the supernatant of β-Gal restimulated splenocytes. Thus, only splenocytes of mice receiving β-Gal with the adjuvant system c-di-AMP/alum secreted statistically significantly higher levels of Th1 (IFN-γ, IL-2), Th2 (IL-4, IL-5, IL-10, IL-13), and Th17 (IL-17A, IL-22) cytokines compared to the group receiving β-Gal alone with *p* < 0.05 (Figure 5). However, no significant differences have been observed for TNF-α. Interestingly, the addition of c-di-AMP in the vaccine formulation with alum evokes a Th17 polarization of the lymphocytes following i.m. vaccination while c-di-AMP and alum stimulated only marginal IL-17 production if any at all (Figure 5). This is remarkable, since it is known already that c-di-AMP stimulates a strong IL-17 production only when applied by mucosal routes (Ebensen et al., 2011, 2017; Mittal et al., 2015). Taken together, the combination of c-di-AMP and alum seems to not only sustain the Th2 response stimulated by β-Gal but, in addition, strengthen the β-Gal-specifc Th1 and Th17 responses.

## Discussion

In contrast to most medications, vaccines are administered to large populations of more or less healthy persons. Moreover, also infants, children and immunocompromised individuals are target subpopulations, making no or only low potential risks or side-effects essential for vaccines (Di Pasquale et al., 2016). In order to increase vaccine safety, recent research has focused on so-called subunit vaccines. In contrast to common vaccine formulations, these vaccines usually consist only of microbial components. Unfortunately, the increased safety profile is at the expense of the vaccine efficacy, as pure antigens typically are only poorly immunogenic. Therefore, subunit vaccines essentially have to include antigen delivery systems or adjuvants (Ebensen and Guzman, 2008).

Adjuvants are key elements in both prophylactic as well as therapeutic vaccines since they can not only improve the strength of antigen-specific immune responses but also modify these responses according to the specific needs (Di Pasquale et al., 2016). However, one of the challenges in adjuvant development is to balance efficacy and safety in order to stimulate immunity (Batista-Duharte et al., 2018).

Besides a handful of adjuvants such as MF59, monophosphoryl lipid A (MPL A), AS03, AF03 or virosomes, only aluminum-based adjuvants (e.g., AS04) continue to be used worldwide since over 90 years. The main forms of aluminum adjuvant used in parenteral administered vaccines are aluminum hydroxide and aluminum phosphate (He et al., 2015). In addition to the beneficial aspects of alum, there are also some limiting effects such as induction of adverse reactions and preferential priming of Th2-type immune responses (He et al., 2015). In this regard, novel adjuvants including also mixtures of adjuvants (adjuvant systems) have opened the door for the development of vaccines with improved safety and efficacy profiles against emerging and/or re-emerging pathogens (Garcon and Di Pasquale, 2017). Thus, adjuvant combinations targeting different pattern-recognition receptors (PRR), both endosomal and intracellular, enhance antigen-specific immune responses and/or direct them toward the response of need (e.g., cytotoxic or mucosal response) (Gutjahr et al., 2016). For example, the combination of the Toll-like receptor (TLR) agonist MPL A adsorbed on aluminum salts (AS04^TM^, GlaxoSmithKline) results in the stimulation of increased production of antigen-specific antibodies and an enhanced cell-mediated response by causing a local and temporary cytokine response (Reed et al., 2009; Garçon et al., 2011; Del Giudice et al., 2018). Nevertheless, no efficient antigen-specific Th1 and cytotoxic T cell responses are stimulated.

Therefore, the aim of the present work was the exploration of the great potential of c-di-AMP acting as a parenteral adjuvant in an adjuvant system combined with alum and the model antigen β-Gal to overcome the limitations of alum, since c-di-AMP was shown to stimulate strong Th1 and cytotoxic responses even when included in adjuvant combinations (Mittal et al., 2015; Ebensen et al., 2017; Matos et al., 2017; Sanchez Alberti et al., 2017; Schulze et al., 2017a; Temizoz et al., 2018). c-di-AMP binds to the transmembrane protein STING (stimulator of IFN genes) thereby activating the TBK3-IRF3 signaling pathway, subsequently triggering the production of type I IFN and TNF (McWhirter et al., 2009; Burdette et al., 2011; Shu et al., 2012). This in turn, results in strong adaptive immune responses.

In line with previously obtained results, co-administration of β-Gal with either alum or c-di-AMP alone stimulated strong humoral β-Gal-specific immune responses (Ebensen et al., 2011; McKee and Marrack, 2017). However, when immunizing mice with the combination of alum and c-di-AMP, the detected IgG titers were further increased compared to those observed in sera of mice receiving alum or c-di-AMP alone (factor 2.7 and 6.4, respectively). In order to evaluate if the combination of alum and c-di-AMP will also have a beneficial effect on antigen-specific cellular responses, we analyzed the proliferative capacity as well as the cytokine profiles of splenocytes of animals vaccinated i.m. with β-Gal alone or in combination with c-di-AMP, alum or the adjuvant system c-di-AMP/alum. Similar to the observed humoral responses, the adjuvant system also stimulated increased antigen-specific cellular responses as indicated by the strong proliferative capacity already at relatively low doses of β-Gal antigen compared to the formulations encompassing only a single adjuvant. In addition, the observed cytokine profile stimulated following vaccination reveals the characteristic of cell-mediated effector functions. Thus, splenocytes recovered from mice vaccinated with the adjuvant system c-di-AMP/alum plus β-Gal were found to produce enhanced levels of the Th1 cytokines IFN-γ and IL-2, the Th2 cytokines IL-4, IL-5, IL-10 and IL-13, and the Th17 cytokine IL-17, whereby the Th1/Th2 ratio was shifted toward Th1. In general, groups receiving either c-di-AMP or alum alone as adjuvant gave significantly lower numbers of cytokine-producing cells and cytokine titers compared to the group receiving the adjuvant combination. The observed cellular responses were confirmed by the obtained humoral immune responses, such as IgG titer and IgG subclass profiles. Hence, in line with previous studies, mice immunized with β-Gal co-administered with c-di-AMP showed a balanced production of IgG1 and IgG2a, correlating with a balanced Th1/Th2 response (Libanova et al., 2010; Ebensen et al., 2011; Mittal et al., 2015). The same is true for mice receiving the adjuvant system c-di-AMP/alum which showed a balanced IgG1/IgG2a ratio of approximately three, while alum alone promoted a Th2-biased response visualized by a IgG1/IgG2a ratio greater than 40.

Interestingly, the combination of alum with c-di-AMP also resulted in the stimulation of enhanced levels of IL-10, which was shown to block Th1 responses. Thus, when Oleszycka and co-workers immunized mice using alum as adjuvant, increased IL-10 titers were observed but only limited Th1 responses. In contrast, when they immunized IL-10 deficient mice using alum, increased Th1 responses have been obtained suggesting an inhibitory effect of IL-10 on Th1 cells (Oleszycka et al., 2018). However, previous findings also showed that induction of IL-10, which promotes IgA switch, displays broad anti-inflammatory properties (Lamm and Phillips-Quagliata, 2002) and is involved in self-regulation of Th1 responses (Jankovic et al., 2010). Thus, the strong IL-10 titers observed in mice vaccinated with β-Gal co-administered with the adjuvant system c-di-AMP/alum might simply reflect the necessity of self-regulation in order to restrain the stimulated antigen-specific Th1 immune response and prevent pathology (Ng et al., 2013). Likewise, vaccination using the adjuvant system c-di-AMP/alum also stimulated elevated levels of IL-17. This is in line with previous observations showing that alum-adjuvanted vaccines stimulated efficient immunity based on Th17 responses, which could even be improved when alum was combined with other adjuvants resulting in the induction of Th1/Th17 responses (Ross et al., 2013; Bagnoli et al., 2015). Interestingly, while c-di-AMP has been shown to facilitate Th17 polarization when administered by mucosal routes only marginal, if any at all, IL-17 production is stimulated when c-di-AMP is administered parenteral (Mittal et al., 2015; Ebensen et al., 2017). Therefore, the combination of alum with c-di-AMP applied by i.m. route seems to overcome this confinement.

Nevertheless, although the incorporation of c-di-AMP efficiently stimulates Th1 responses, the adjuvant system c-di-AMP/alum seems to be insufficient in compensating the inadequate stimulation of CD8+ T cells by alum as indicated by similar levels of IFNγ+ CD8-peptide-specific T cells in mice immunized using alum and the combination of alum and c-di-AMP, respectively. Instead, alum seems to inhibit the stimulation of CD8+ T cells by c-di-AMP. Further experiments need to be performed investigating whether higher concentrations of c-di-AMP would invert this effect.

Taken together, functionally distinct effector CD4+ T helper cell subsets are characterized by the secretion of distinct cytokine profiles. By this, our immune system is able to efficiently combat rapidly evolving and spreading pathogens infecting the host (Kara et al., 2014). Thus, while Th2 cells are critical for effective humoral immunity, by facilitating affinity maturation and class switch of the antigen-specific antibodies necessary for eradication of extracellular pathogens, Th1 cells are important for cell-mediated defense mechanisms helping to eliminate intracellular pathogens such as viruses (Chen and Cerutti, 2010). However, research in vaccine development seems to reveal that a single adjuvant will hardly be able to cope with all the foreseeable requirements in the field of infectious diseases. Thus, the development of new adjuvants increasing the portfolio of immunomodulatory molecules which allow the formulation of most effective vaccines tailored for the specific needs is essential. This tool box for vaccine developers can be even extended by combining adjuvants with different mechanisms of action. Moreover, adjuvant combination can also increase the safety profile of a certain vaccine, as in the case of alum, for example, the likelihood of connected adverse side effects is reduced by restraining immune reactions and diminishing the risk of immuno-pathological outcomes (Didierlaurent et al., 2009).

In this regard, our results demonstrate that the adjuvant system c-di-AMP/alum generates conditions sufficient for stimulating both humoral and cellular immune responses. Moreover, the combination of alum with c-di-AMP not only strengthens the stimulated antigen-specific immune responses but also modulates them in the direction of a Th1 response.

## Ethics Statement

All animal experiments in this study were approved by the institutional ethical board and have been performed with ethical agreement by the local government of Lower Saxony (Germany) with the No. 33.42502 13/1281.

## Disclosure

CAG and TE are named as inventors in a patent application covering the use of c-di-AMP as adjuvant (PCT/EP 2006010693). This does not alter our adherence to the Frontier Science policies on sharing data.

## Author Contributions

TE and KS conceived and designed the study. TE, KS, and SD performed the experiments. analyzed and interpreted the data, and wrote the manuscript. BP contributed to the writing and proofreading of the manuscript. CG gave scientific advice and supervised the work.

### Conflict of Interest Statement

The authors declare that the research was conducted in the absence of any commercial or financial relationships that could be construed as a potential conflict of interest.
